# Expression of visfatin in gingival crevicular fluid and gingival tissues in different periodontal conditions: a cross-sectional study

**DOI:** 10.1186/s12903-024-04299-2

**Published:** 2024-05-02

**Authors:** Kang Xiao, Ling Chen, Yudian Mao, Han Bao, Weirong Chen, Xiang Li, Yun Wu

**Affiliations:** 1 Fujian Key Laboratory of Oral Diseases & Fujian Provincial Engineering Research Center of Oral Biomaterial & Stomatological Key Lab of Fujian College and University, School and Hospital of Stomatology, Fujian Medical University, Fuzhou, China; 2 Institute of Stomatology & Research Center of Dental and Craniofacial Implants, School and Hospital of Stomatology, Fujian Medical University, Fuzhou, China; 3 Stomatological Center, The First Affiliated Hospital, Fujian Medical University, Fuzhou, China; 4 Stomatological Center, National Regional Medical Center, Binhai Campus of the First Affiliated Hospital, Fujian Medical University, Fuzhou, 350212 China

**Keywords:** Visfatin, Periodontitis, Biomarker, Gingival crevicular fluid

## Abstract

**Background:**

Studies have shown that visfatin is an inflammatory factor closely related to periodontitis. We examined the levels of visfatin in gingival crevicular fluid (GCF) and gingival tissues under different periodontal conditions, in order to provide more theoretical basis for exploring the role of visfatin in the pathogenesis of periodontitis.

**Methods:**

We enrolled 87 subjects, with 43 in the chronic periodontitis (CP) group, 21 in the chronic gingivitis (CG) group, and 23 in the periodontal health (PH) group. Periodontal indexes (PD, AL, PLI, and BI) were recorded. GCF samples were collected for visfatin quantification, and gingival tissues were assessed via immunohistochemical staining.

**Results:**

Visfatin levels in GCF decreased sequentially from CP to CG and PH groups, with statistically significant differences (*P* < 0.05). The CP group exhibited the highest visfatin levels, while the PH group had the lowest. Gingival tissues showed a similar trend, with significant differences between groups (*P* < 0.001). Periodontal indexes were positively correlated with visfatin levels in both GCF and gingival tissues (*P* < 0.001). A strong positive correlation was observed between visfatin levels in GCF and gingival tissues (rs = 0.772, *P* < 0.001).

**Conclusion:**

Greater periodontal destruction corresponded to higher visfatin levels in GCF and gingival tissues, indicating their potential collaboration in damaging periodontal tissues. Visfatin emerges as a promising biomarker for periodontitis and may play a role in its pathogenesis.

**Supplementary Information:**

The online version contains supplementary material available at 10.1186/s12903-024-04299-2.

## Background

Severe periodontitis ranks as the sixth most common human disease, exhibiting a high prevalence worldwide. It is one of the chronic oral diseases that pose a significant threat to human health and thus necessitates prevention and treatment measures [1]. Chronic periodontitis is an enduring infectious disease resulting from plaque biofilm, which adversely affects the periodontal support tissues [2]. Traditional diagnostic methods, such as assessing periodontal pocket depth, clinical attachment level, bleeding on probing, and radiological examination, offer valuable insights for evaluating the severity of periodontal disease. However, these methods have limitations in assessing the current activity levels of the disease [3]. In periodontology, biomarkers furnish supplementary information beyond routine clinical and radiological examinations [4].

Adipose tissue serves as an energy storage organ with metabolic activity and has the ability to secrete adipokines that play a role in immune regulation. Notable adipokines, including visfatin, adiponectin, leptin, tumor necrosis factor-α, and interleukin-1β (IL-1β), are widely involved in systemic immune and inflammatory responses [5, 6]. Visfatin is an adipokine that can bind to insulin receptors, activate insulin receptor signaling pathways, and regulate glucose anabolism [7]. Given visfatin’s diverse biological properties, which encompass functioning as enzymes, growth factors, and pro-inflammatory cytokines, it has been associated with a range of conditions within the body. These include obesity [8], insulin resistance and diabetes [7–9], atherosclerosis [10], cardiovascular disease [11], rheumatoid arthritis [12], and other diseases.

In recent years, visfatin’s involvement in the regulation of inflammatory responses has been found to play a crucial role in the pathogenesis of periodontitis [13, 14]. Visfatin exhibits significant upregulation in human fibroblasts and exerts influence on the inflammatory response by modulating the levels of cyclooxygenase-2, matrix metalloproteinase-1(MMP-1), and matrix metalloproteinase-3 (MMP-3). It serves as a key mediator of periodontal inflammation and alveolar bone destruction [15]. Gingival crevicular fluid (GCF) is a combination of physiologic fluid originating from the gingival vascular plexus and serum inflammatory exudate [16, 17]. Specific biomarkers within GCF can distinguish between periodontal health and disease conditions, enabling their application in the diagnosis, prognosis, and management of periodontal disease [18–21]. Chronic gingivitis is characterized by gingival inflammation without the loss of periodontal supportive tissues [22]. One study revealed that patients in the periodontitis group had higher levels of GCF and serum visfatin compared to patients in the gingivitis group and healthy controls. Furthermore, the gingivitis group exhibited higher visfatin levels than healthy controls. Salivary visfatin levels were significantly elevated in patients with periodontitis, and a significant positive correlation was observed between visfatin levels and clinical attachment level [23]. It was also demonstrated that GCF and serum visfatin levels decreased following periodontal treatment, with visfatin levels correspondingly decreasing as periodontal conditions improved [24]. However, few studies have explored the correlation with visfatin expression levels in gingival tissues. Some researchers collected gingival tissue specimens from individuals with periodontal health and patients with periodontitis for immunohistochemical staining. The results indicated that gingival tissues of patients with periodontitis displayed widespread and strong visfatin expression, diffusely distributed within epithelial cells, human gingival fibroblasts, cytoplasm, endothelial cells, and the intercellular substance of human gingival fibroblasts, albeit weakly expressed [25]. This study suggested that localized visfatin expression in gingival tissues may be associated with the extent of periodontal inflammation.

In this study, we introduced an innovative approach by simultaneously collecting both GCF and gingival tissue specimens from the same periodontal site under varying periodontal conditions. We conducted separate assessments of their visfatin expression levels to explore the correlation between visfatin expression and the severity of periodontal inflammation. The primary objective of this study is to establish a theoretical foundation for utilizing visfatin as a potential biomarker for periodontitis.

## Methods

### Study population

For this study, we selected patients admitted to the Stomatological Center of the First Affiliated Hospital of Fujian Medical University between October 2021 and June 2022. The study received approval from the Ethics Committee of the First Affiliated Hospital of Fujian Medical University (Aprove No: MRCTA, ECFAH of FMU [2021]155), and consent was obtained from all participating subjects.

#### Inclusion criteria

Participants of Han nationality, aged 18–70 years; Maintained consistent oral hygiene and dietary habits for at least 3 months; No history of prior periodontal treatment.

#### Exclusion criteria

Prolonged use of antibiotics, hormones, or immune-related medications; pregnancy or lactation; smoking habits; presence of systemic diseases that affect periodontal health, such as diabetes or autoimmune diseases.

In accordance with the aforementioned criteria and the classification criteria of the American Academy of Periodontology in 1999 [22], a total of 87 cases were included in the study. There were 43 cases in the chronic periodontitis group (CP group), 21 cases in the chronic gingivitis group (CG group), and 23 cases in the periodontal health group (PH group).

### Sample collection sites

#### CP group

Teeth with periodontitis and no retentive value.

#### CG group

Third molars with no retentive value.

#### PH group

Third molars with no retentive value or teeth requiring gingivectomy during crown lengthening surgery.

### Clinical examination and data collection

General information about the subjects was collected, including gender, age, height, weight, and body mass index (BMI). Periodontal examinations are performed by the same professionally trained periodontist. A self-repeated measurement consistency test, requiring a kappa value > 0.95, is performed prior to the examination. Periodontal indexes, including probing depth (PD), attachment loss (AL), plaque index (PLI) [26], and bleeding index (BI) [27], were to be recorded for each sample collection site in each group. Using the Williams periodontal probe, six sites (buccal: distal, central, mesial; and lingual: distal, central, mesial) on the selected teeth were checked. The average values of PD, AL, PLI, and BI were calculated.

### Collection and detection of GCF

To collect GCF, a sterile cotton ball was used to isolate the teeth from moisture. Carefully remove supragingival plaque, blowing gently with an air gun from the root to the crown of the tooth, which dries the surface of the tooth without blowing away the GCF. Absorbent paper points (DIA-ISO 0.02 030, Beijing Dayading Medical Appliance Co. LTD, China) were inserted into the mesial and distal gingival sulcus on the buccal side of the tooth. These paper points were removed after 30 s and placed in a sterile Eppendorf tube. If the absorbent paper points were contaminated with blood or saliva, they were discarded and resampled after 10 min. The paper points were sealed in Eppendorf tubes and stored in a -80 °C refrigerator.

Visfatin levels in GCF were measured using a double-antibody one-step sandwich enzyme-linked immunosorbent assay (ELISA), following the instructions of the Human Visfatin ELISA Kit (Shanghai Qiaodu Biotechnology Co. LTD, China). Sensitivity is a minimum detection concentration of less than 1.0 ng/mL. Take out the required slats from the aluminum foil pouch after equilibrating at room temperature for 20 min, set up standard and sample wells, add 50 of different concentrations of standards to each standard well; add 10 µL of samples to be tested to the sample wells, then add 40 µL of sample dilution, and do not add to the blank wells; except for the blank wells, add 100 µL of horseradish peroxidase (HRP)-labeled detection antibody to each of the standard wells and sample wells, and seal the reaction wells with a plate-sealing membrane. The reaction wells were sealed with plate sealing membrane and incubated at 37℃ for 60 min; the liquid was discarded and patted dry on blotting paper, each well was filled with washing solution and left to stand for 1 min, the washing solution was shaken off and the blotting paper was patted dry, and the plate was washed for 5 times; 50 µL of substrate A and B were added to each well, and the plate was incubated for 15 min at 37℃ away from light, and finally 50 µL of termination solution was added to each well. The absorbance value was measured with a microplate reader at a wavelength of 450 nm. A linear regression curve was constructed from the standard, and the concentration of each sample was calculated based on the curve equation.

### Gingival tissues collection, immunohistochemical staining, and quantitative analysis

After GCF collection and periodontal examination, tooth extraction was performed. Small amounts of gingival tissue were cut from the buccal side of the extraction socket. These tissue samples were approximately 3~4 mm in length, and 1.5 mm in thickness, and included a full layer of epithelial and connective tissue. Gingival tissue samples were promptly fixed with 10% neutral formalin, embedded in paraffin, and made into 4 μm sections.

Paraffin sections were dewaxed and hydrated, and antigens were repaired with EDTA solution. An endogenous peroxidase blocker was added, incubated for 10 min, and rinsed with phosphate-buffered saline (PBS) (pH = 7.4). The primary antibody (Recombinant Anti-Visfatin antibody [EPR21980], Abcam, UK) was added at a 1:2000 dilution ratio, incubated for 60 min, and rinsed with PBS. Secondary antibody (enzyme-labeled goat anti-mouse/rabbit IgG polymer) was applied, incubated for 15 min, and rinsed with PBS. Diaminobenzidine color development solution was added and incubated for 3 min. Finally, the sections were rinsed with distilled water, re-stained with hematoxylin for 1 min, dehydrated, transparentized, and sealed. The experiment was conducted at an incubation temperature of 37 °C, with consistent steps to ensure that different sections were treated in the same experimental environment and following the same procedure.

Microscope working conditions and shooting parameters were consistent. Five random fields of view (400 ×) were selected in three gingival tissue areas: full layer, epithelial layer, and connective tissue layer. An experimenter who was unaware of the section grouping captured all sections at once. Software (ImageJ, National Institutes of Health, USA) was used to quantitatively analyze the images. The gray value and optical density were converted and calibrated, and the average optical density (AOD) value of the positively stained area in the images was calculated. The average AOD values from the five fields of view represented the staining intensity of the area, indicating the expression level of the target protein [28].

### Statistical analysis

Statistical analysis was carried out using statistical software (SPSS 26.0, IBM, USA). Mean ± standard deviation (Mean ± SD) was used to express the data’s central tendency and dispersion. Dichotomous variables were compared using the χ2 test. The Mann-Whitney U test was employed to compare measurement data between the two groups. The Kruskal-Wallis H test and Bonferroni correction method were used for multiple post hoc comparisons. *P* < 0.05 was considered statistically significant. Correlations between variables were assessed using Spearman’s rank correlation analysis.

## Results

### Comparison of general information and periodontal indexes

When comparing the general information of the CP, CG, and PH groups, the CP group’s age (49.14 ± 9.49 years) was significantly higher than that of the CG group (25.29 ± 5.07 years) and the PH group (26.87 ± 4.58 years) (*P* < 0.001). No statistically significant differences were observed in gender composition ratio, height, weight, or BMI among the three groups (*P* > 0.05). During the selection of study subjects, the significant age difference between the CP, CG, and PH groups, where the CG and PH groups were predominantly younger, indicates a potential confounding effect of age on the study results. However, there are no studies that show a direct and specific correlation between age and visfatin. The study population was carefully selected to minimize the influence of confounding factors, ensuring balanced baseline characteristics among the three groups and resulting in reliable and comparable results.

Regarding the periodontal indexes in the CP, CG, and PH groups, the results indicated that PD, AL, PLI, and BI in the CP group were significantly higher than in the CG and PH groups, with statistically significant differences (*P* < 0.001). PD and BI in the CG group were also higher than in the PH group, with statistically significant differences (*P* < 0.002). While PLI in the CG group was higher than in the PH group, the difference was not statistically significant (*P* > 0.05) (Table 1).

**Table 1:** Comparison of general information and periodontal indexes in different periodontal conditions

	CP group	CG group	PH group	χ²/K	*P*
	*n* = 43	*n* = 21	*n* = 23		
Male (n)	21	10	11	0.011	0.995
Female (n)	22	11	12		
Age (year)	49.14 ± 9.49	25.29 ± 5.07	26.87 ± 4.58	62.455	< 0.001*
Height (m)	1.66 ± 0.05	1.62 ± 0.06	1.63 ± 0.08	1.918	0.153
Weight (Kg)	65.19 ± 5.90	62.71 ± 4.83	62.48 ± 7.50	1.949	0.149
BMI(Kg/m^2^)	23.74 ± 1.96	23.74 ± 2.07	23.32 ± 1.47	0.542	0.763
PD (mm)	5.30 ± 1.70	3.41 ± 1.16	2.11 ± 0.51	52.891	< 0.001*
AL (mm)	5.69 ± 1.97	0.00 ± 0.00	0.00 ± 0.00	74.102	< 0.001*
PLI	2.40 ± 0.82	1.29 ± 0.72	0.65 ± 0.78	42.050	< 0.001*
BI	3.63 ± 0.54	1.95 ± 1.32	0.00 ± 0.00	64.350	< 0.001*

Data presented as Mean ± SD

* Significant (*p* < 0.05)

### Comparison of visfatin expression in GCF and gingival tissues

**Fig. 1:**
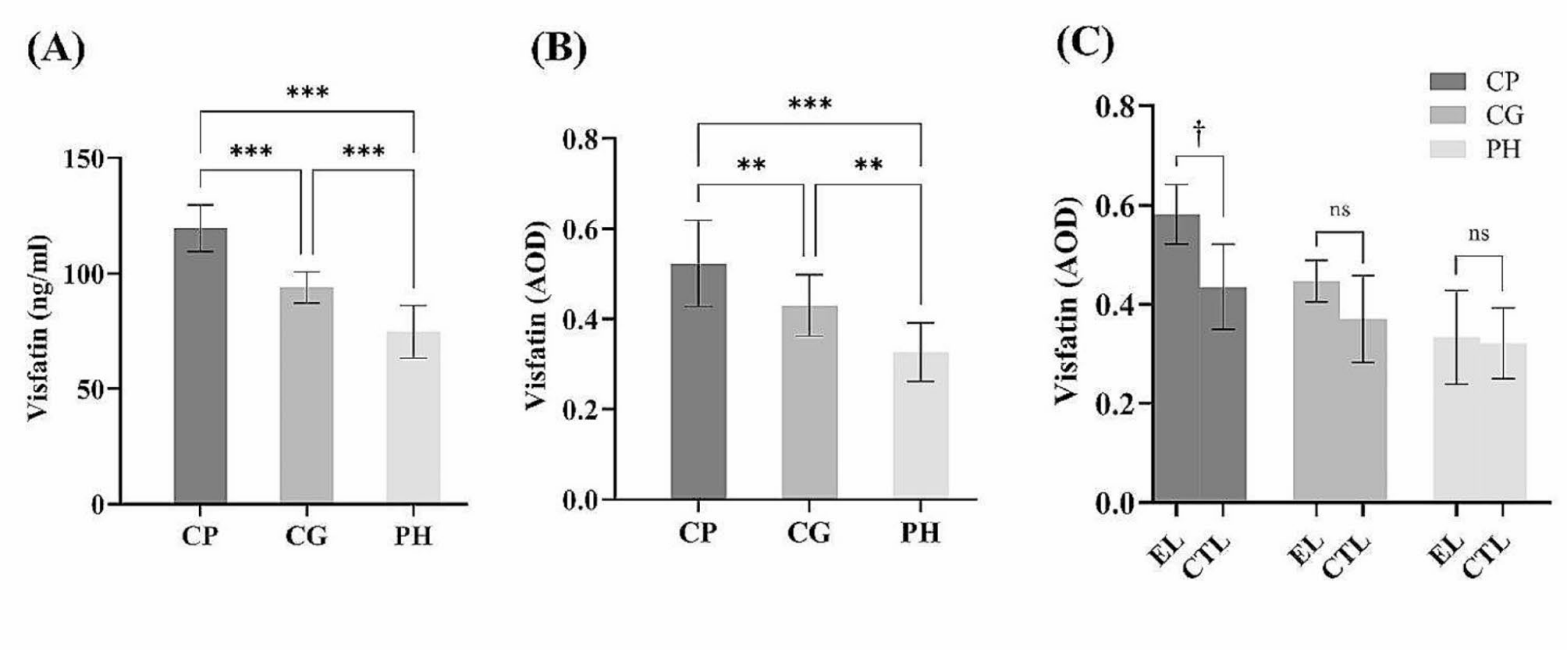
Comparisons of visfatin in GCF and gingival tissues. (**A**): Multiple comparisons of visfatin in GCF. (**B**): Multiple comparisons of visfatin in gingival tissues. (**C**): Comparison of visfatin expression in epithelial layer and connective tissue layer of gingival tissues. EL: Epithelial layer; CTL: Connective tissue layer. Adjusted significance: * *P* < 0.0332; ** *P* < 0.0021; **** *P* < 0.0001. † :Significant (*p* < 0.05); ns: not significant

Visfatin levels in GCF for the CP, CG, and PH groups were 119.58 ± 10.15 ng/mL, 93.85 ± 6.81 ng/mL, and 74.64 ± 11.37 ng/mL, respectively, and these differences were statistically significant (*P* < 0.001). Statistically significant differences were also observed when comparing the three groups in pairs (*P* < 0.001) (Fig. 1A).

**Fig. 2:**
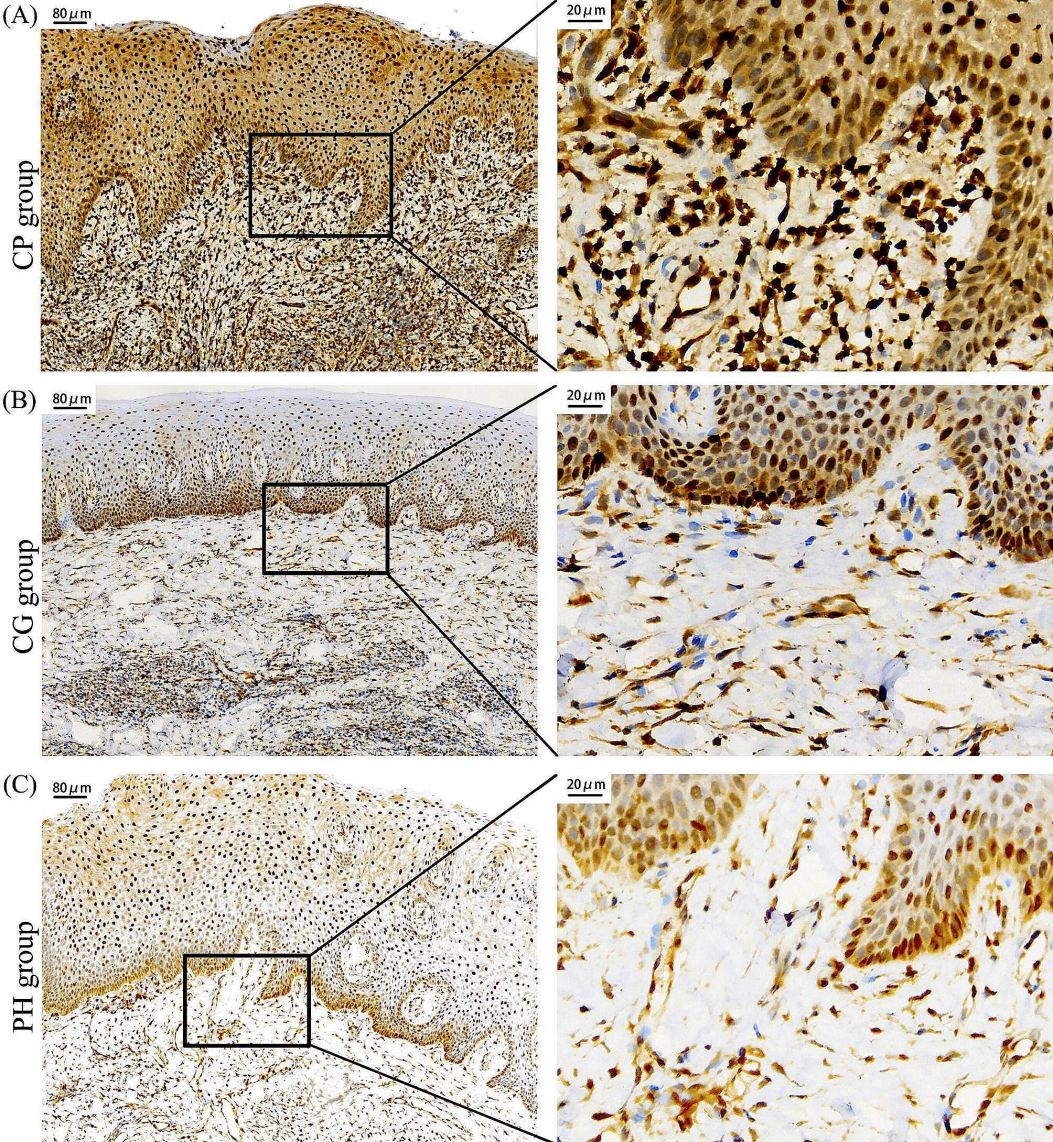
Expression of visfatin in gingival tissues of different periodontal conditions. (**A**) CP group. (**B**) CG group. (**C**) PH group

Immunohistochemical sections viewed under a light microscope revealed that visfatin was diffusely expressed in all layers of the epithelium, with the exception of cells in the stratum corneum and part of the granular layer adjacent to the stratum corneum. Visfatin staining was particularly intense in the basal layer. In the connective tissue layer, visfatin expression was observed in fibroblasts, endothelial cells, lymphocytes, and the intercellular matrix (Fig. 2).

When comparing different periodontal conditions, the AOD values for the whole-layer field of view in the CP, CG, and PH groups were 0.52 ± 0.10, 0.43 ± 0.07, and 0.33 ± 0.06, respectively. Significant differences were observed among the three groups, as well as in multiple comparisons (*P* < 0.001) (Fig. 1B).

Comparing different areas of expression, the AOD values for the epithelial layer and connective tissue layer in the CP group were 0.58 ± 0.06 and 0.43 ± 0.09, respectively. In the CG group, the AOD values for the epithelial layer and connective tissue layer were 0.45 ± 0.04 and 0.37 ± 0.09, respectively. In the PH group, the AOD values for the epithelial layer and connective tissue layer were 0.33 ± 0.09 and 0.32 ± 0.07, respectively. In the CP group, the epithelial layer had a higher AOD value than the connective tissue layer, and this difference was statistically significant (*P* < 0.05). However, in the CG and PH groups, the AOD values for the epithelial layer were higher than those for the connective tissue layer, but the differences were not statistically significant (*P* > 0.05) (Fig. 1C). This suggests that the gingival tissues of patients in the CG and PH groups exhibited more balanced levels of visfatin expression between the epithelial and connective tissue layers.

### Correlation between periodontal index and visfatin expression

Spearman’s correlation analysis was employed to assess the relationships between periodontal indexes (PD, AL, PLI, and BI), as well as the levels of visfatin in GCF and gingival tissues (Table 2). The results indicated that PD, AL, PLI, and BI were positively correlated with visfatin levels in GCF, with a statistically significant correlation (*P* < 0.001). Similarly, PD, AL, PLI, and BI were positively correlated with visfatin levels in gingival tissues, also showing a statistically significant correlation (*P* < 0.001). The correlation coefficients between visfatin levels in GCF and those in gingival tissues had a correlation coefficient of rs = 0.772 (*P* < 0.001).

**Table 2:** Correlation between periodontal indexes and expression of visfatin

		PD	AL	PLI	BI	Visfatin(GCF)	Visfatin(Gingival)	
		(mm)	(mm)			(ng/ml)	(AOD)	
Visfatin(GCF)	rs	0.920*	0.870*	0.736*	0.835*	1.000	0.772*	
(ng/ml)	p	< 0.001	< 0.001	< 0.001	< 0.001	-	< 0.001	
Visfatin(Gingival)	rs	0.757*	0.694*	0.534*	0.700*	0.772*	1.000	
(AOD)	p	< 0.001	< 0.001	< 0.001	< 0.001	< 0.001	-	

*Correlation is significant at the 0.01 level (2-tailed)

## Discussion

The interaction between periodontal pathogenic bacteria and the host immune-inflammatory response is pivotal in the development and progression of periodontitis. Periodontal pathogens trigger the local secretion of inflammatory mediators and activate inflammatory signaling pathways in host cells to combat the pathogens, but this response also leads to bone resorption and collagen breakdown [29]. Periodontal pockets, clinical attachment loss, and gingival inflammation are key clinical manifestations of periodontitis, and the periodontal indices PD, AL, PLI, and BI objectively reflect the severity of periodontal inflammation [30, 31]. In this study, each periodontal index was consistently higher in the CP group compared to the CG and PH groups. PD and BI were also higher in the CG group compared to the PH group. This aligns with the expected order of periodontal destruction among the three groups.

Visfatin plays a role in this complex interaction of periodontal tissue destruction. Visfatin expression in GCF is strongly correlated with the periodontal condition. For example, Pradeep et al. [32] showed that both localized visfatin levels in GCF and serum visfatin levels increased with disease severity, from the healthy control group to the gingivitis group, and finally to the periodontitis group. Visfatin levels in GCF and serum in the periodontitis group were positively correlated with periodontal indexes such as PD and AL. In this experiment, as the degree of periodontal lesions increased in the PH, CG, and CP groups, visfatin levels also increased significantly. There was a positive correlation between visfatin levels in GCF and periodontal indexes (PD, AL, PLI, BI), consistent with the findings of other studies [23, 24, 32]. GCF and serum visfatin levels increased in both periodontitis and gingivitis patients compared to healthy controls, and the mean salivary visfatin levels also increased with the severity of periodontal tissue destruction [33, 34]. Non-surgical periodontal treatment significantly improved the periodontal conditions of patients with periodontitis, leading to reduced PD, increased clinical attachment levels, and decreased release of inflammatory mediators from local tissues. After periodontal treatment, GCF and serum visfatin levels were substantially reduced, changing in parallel with the degree of periodontal destruction and influenced by periodontal interventions [23]. In summary, GCF and serum visfatin levels were closely associated with the severity of periodontal inflammation, and the changes in both were consistent.

Visfatin expression levels in gingival tissues are also closely linked to periodontal conditions. Elevated visfatin expression has been observed in inflamed gingival tissues in both humans and mice [15]. Immunohistochemical staining revealed higher levels of visfatin in aggressive and chronic periodontitis gingival tissues compared to healthy gingival tissues, with no difference between aggressive and chronic periodontitis groups [35]. Visfatin expression was significantly higher in gingival tissues of periodontitis patients compared to those of healthy individuals, suggesting that local synthesis of visfatin in inflamed gingival tissues may contribute to increased visfatin levels in gingival tissues and serum [36]. In this experiment, we compared the expression levels of visfatin in the entire field of view of gingival tissues. Notably, the intensity of visfatin expression progressively weakened in the CP, CG, and PH groups. Furthermore, our correlation analysis revealed a positive association between periodontal indexes, including PD, AL, PLI, BI, and visfatin levels within gingival tissues. As the severity of periodontal destruction increased, so did the expression of visfatin in gingival tissues.

Additionally, our correlation analysis unveiled a positive relationship between visfatin levels in GCF and visfatin levels in gingival tissues. GCF, being a complex mixture originating from serum and periodontal tissues, is widely acknowledged as a reflection of the inflammatory state of periodontal tissues [37]. This finding leads us to speculate that GCF might, in part, originate from the gingival tissue itself, signifying its role in the local inflammatory context.

The distribution sites of visfatin expression in gingival tissues vary across different periodontal conditions. Studies have shown that visfatin is strongly expressed in all layers of gingival tissue in patients with periodontitis, with diffuse distribution in the epithelial layer and expression in fibroblasts, endothelial cells, and intercellular matrix in the connective tissue layer [35, 36]. In healthy gingiva, visfatin is mainly limited to the basal and parabasal layers of the epithelium, with lower intensity elsewhere and weak or no expression in the connective tissue layer [25, 35]. Some studies have reported variations, such as no positive staining in the basal layer of gingival epithelium in gingivitis and healthy control patients. A small amount of visfatin was expressed in the connective tissue layer of gingival tissues in patients with gingivitis, while no expression of visfatin was found in the connective tissue layer of gingival tissues in healthy individuals [36]. The distribution of visfatin expression within gingival tissues exhibits slight variations. The results of this experiment indicate that in the CP group, the level of visfatin in the epithelial layer surpasses that in the connective tissue layer. In contrast, the CG and PH groups display a balanced expression of visfatin in both the epithelial and connective tissue layers, with no significant differences. Numerous studies mentioned in the literature consistently report a higher expression of visfatin in the epithelial layer compared to the connective tissue layer. This phenomenon was also observed within the CP group in our study. The authors posit that the observed differences in expression distribution across a limited number of studies may arise from variations in the antibody products utilized. Moreover, the elevated level of visfatin in the epithelial layer compared to the connective tissue layer in the CP group may be attributed, in part, to certain epithelial cells demonstrating a greater propensity or capacity to produce visfatin. Additionally, it could be influenced by the anatomical positioning of the gingival and sulcus epithelium, making them more susceptible to stimulation from the oral flora, particularly periodontal pathogenic bacteria, potentially leading to enhanced visfatin production.

Periodontal pathogenic bacteria like *Porphyromonas gingivalis* and *Clostridium nucleatum*, as well as pro-inflammatory cytokines like IL-1β, can stimulate visfatin synthesis in periodontal ligament cells and gingival fibroblasts, with increased synthesis under inflammatory and infectious conditions [36, 38]. Visfatin mediates the inflammatory response by activating nuclear factor-κB and phosphatidylinositol trihydroxykinase signaling pathways, which inhibit neutrophil apoptosis [39]. Visfatin may play a role in periodontitis by upregulating MMP-1 and chemokine-2 in periodontal ligament cells [40]. It can upregulate pro-inflammatory cytokines and matrix metalloproteinases in various cell types, leading to connective tissue and periodontal bone loss [25]. Visfatin plays a crucial role in the resorptive remodeling of alveolar bone and is increased in inflammatory diseases involving bone resorption, such as rheumatoid arthritis and osteoarthritis [41, 42]. Combined with the experimental results, it can be speculated that periodontal pathogenic bacteria might damage periodontal tissues through the upregulation of visfatin expression in local gingival tissues and GCF, further regulating various inflammatory factors. Periodontal pathogenic bacteria can cause inflammation in periodontal tissues, and periodontal indexes like PD, AL, and BI reflect the severity of periodontal inflammation. Simple gingival inflammation without periodontal pockets and attachment loss can also lead to elevated levels of GCF and visfatin in gingival tissues. The CG group exhibited less severe periodontal inflammation than the CP group, resulting in lower levels of GCF and visfatin in gingival tissues due to reduced exposure to periodontal pathogenic bacteria and their virulence products. More inflammation in periodontal tissues leads to higher visfatin synthesis in GCF and gingival tissues, resulting in a stronger destructive effect.

This study also has limitations. This experiment is a cross-sectional study, which has not yet been able to directly reveal the direct causal relationship and specific mechanisms between visfatin expression levels and periodontal disease. Second, the inclusion and exclusion criteria of this experiment were more stringent, limiting the sample size, but this reduces a variety of potential confounders such as systemic diseases. Also, we only analyzed a single inflammatory factor, which does not yet fully reflect the overall situation of GCF and gingival tissue inflammation in patients with periodontitis. However, the conclusions drawn in this paper, when combined with the literature analysis, provide a basis for the prevention and treatment of periodontitis. In the future, larger samples and longitudinal studies are needed to further investigate the relationship between visfatin and the pathogenesis of periodontitis.

## Conclusions

Visfatin in GCF and gingival tissues appears to collaborate in damaging periodontal tissues, and it plays a role in the pathogenesis of periodontitis. Visfatin serves as a potential biomarker for periodontitis and may contribute to its pathogenesis.

### Electronic supplementary material

Below is the link to the electronic supplementary material.

Supplementary Material 1

## Data Availability

The data that support the findings of this study are available from the corresponding author upon reasonable request.

## Abbreviations

GCF: gingival crevicular fluid
CP: chronic periodontitis
CG: chronic gingivitis
PH: periodontal health
IL-1β: interleukin-1β
BMI: body mass index
PD: probing depth
AL: attachment loss
PLI: plaque index
BI: bleeding index
ELISA: enzyme-linked immunosorbent assay
PBS: phosphate-buffered saline
EL: epithelial layer
CTL: connective tissue layer
