# Supplementary material for: Expression of visfatin in gingival crevicular fluid and gingival tissues in different periodontal conditions: a cross-sectional study
Source: BMC Oral Health. 2024 May 2;24:514. doi: 10.1186/s12903-024-04299-2 (PMC11064311; doi:10.1186/s12903-024-04299-2)
Supplement: Supplementary file 1 — Supplementary Material 1 [file 12903_2024_4299_MOESM1_ESM.docx]

**Title page**

**Title:** Expression of Visfatin in Gingival Crevicular Fluid and Gingival Tissues in Different Periodontal Conditions: A Cross-Sectional Study

**Authors:**

Kang Xiao[MDS]^1,2^,

Ling Chen[MDS]^3,4^,

Yudian Mao[BDS]^1,2^,

Han Bao [BDS]^1,2^,

Weirong Chen[MDS]^3,4^,

Xiang Li[BDS]^3,4^,

Yun Wu [DDS]^3,4,^*

^1^ Fujian Key Laboratory of Oral Diseases & Fujian Provincial Engineering Research Center of Oral Biomaterial & Stomatological Key Lab of Fujian College and University, School and Hospital of Stomatology, Fujian Medical University, Fuzhou, China

^2^ Institute of Stomatology & Research Center of Dental and Craniofacial Implants, School and Hospital of Stomatology, Fujian Medical University, Fuzhou, China

^3^ Stomatological Center, the First Affiliated Hospital, Fujian Medical University, Fuzhou, China

^4^ Stomatological Center, National Regional Medical Center, Binhai Campus of the First Affiliated Hospital, Fujian Medical University, Fuzhou 350212, China.

*Corresponding author: Yun Wu

Email: 1574316628@qq.com

**Abstract**

**Background:** Studies have shown that visfatin is an inflammatory factor closely related to periodontitis. We examined the levels of visfatin in gingival crevicular fluid (GCF) and gingival tissues under different periodontal conditions, in order to provide more theoretical basis for exploring the role of visfatin in the pathogenesis of periodontitis.

**Methods:** We enrolled 87 subjects, with 43 in the chronic periodontitis (CP) group, 21 in the chronic gingivitis (CG) group, and 23 in the periodontal health (PH) group. Periodontal indexes (PD, AL, PLI, and BI) were recorded. GCF samples were collected for visfatin quantification, and gingival tissues were assessed via immunohistochemical staining.

**Results:** Visfatin levels in GCF decreased sequentially from CP to CG and PH groups, with statistically significant differences (P<0.05). The CP group exhibited the highest visfatin levels, while the PH group had the lowest. Gingival tissues showed a similar trend, with significant differences between groups (P<0.001). Periodontal indexes were positively correlated with visfatin levels in both GCF and gingival tissues (P<0.001). A strong positive correlation was observed between visfatin levels in GCF and gingival tissues (rs=0.772, P<0.001).

**Conclusion:** Greater periodontal destruction corresponded to higher visfatin levels in GCF and gingival tissues, indicating their potential collaboration in damaging periodontal tissues. Visfatin emerges as a promising biomarker for periodontitis and may play a role in its pathogenesis.

**Keywords:** visfatin, periodontitis, biomarker, gingival crevicular fluid.

**Background**

Severe periodontitis ranks as the sixth most common human disease, exhibiting a high prevalence worldwide. It is one of the chronic oral diseases that pose a significant threat to human health and thus necessitates prevention and treatment measures^[1]^. Chronic periodontitis is an enduring infectious disease resulting from plaque biofilm, which adversely affects the periodontal support tissues^[2]^. Traditional diagnostic methods, such as assessing periodontal pocket depth, clinical attachment level, bleeding on probing, and radiological examination, offer valuable insights for evaluating the severity of periodontal disease. However, these methods have limitations in assessing the current activity levels of the disease^[3]^. In periodontology, biomarkers furnish supplementary information beyond routine clinical and radiological examinations^[4]^.

Adipose tissue serves as an energy storage organ with metabolic activity and has the ability to secrete adipokines that play a role in immune regulation. Notable adipokines, including visfatin, adiponectin, leptin, tumor necrosis factor-α, and interleukin-1β (IL-1β), are widely involved in systemic immune and inflammatory responses^[5, 6]^. Visfatin is an adipokine that can bind to insulin receptors, activate insulin receptor signaling pathways, and regulate glucose anabolism^[7]^. Given visfatin's diverse biological properties, which encompass functioning as enzymes, growth factors, and pro-inflammatory cytokines, it has been associated with a range of conditions within the body. These include obesity^[8]^, insulin resistance and diabetes^[7-9]^, atherosclerosis^[10]^, cardiovascular disease^[11]^, rheumatoid arthritis^[12]^, and other diseases.

In recent years, visfatin's involvement in the regulation of inflammatory responses has been found to play a crucial role in the pathogenesis of periodontitis^[13, 14]^. Visfatin exhibits significant upregulation in human fibroblasts and exerts influence on the inflammatory response by modulating the levels of cyclooxygenase-2, matrix metalloproteinase-1(MMP-1), and matrix metalloproteinase-3 (MMP-3). It serves as a key mediator of periodontal inflammation and alveolar bone destruction^[15]^. Gingival crevicular fluid (GCF) is a combination of physiologic fluid originating from the gingival vascular plexus and serum inflammatory exudate^[16, 17]^. Specific biomarkers within GCF can distinguish between periodontal health and disease conditions, enabling their application in the diagnosis, prognosis, and management of periodontal disease^[18-21]^. Chronic gingivitis is characterized by gingival inflammation without the loss of periodontal supportive tissues^[22]^. One study revealed that patients in the periodontitis group had higher levels of GCF and serum visfatin compared to patients in the gingivitis group and healthy controls. Furthermore, the gingivitis group exhibited higher visfatin levels than healthy controls. Salivary visfatin levels were significantly elevated in patients with periodontitis, and a significant positive correlation was observed between visfatin levels and clinical attachment level^[23]^. It was also demonstrated that GCF and serum visfatin levels decreased following periodontal treatment, with visfatin levels correspondingly decreasing as periodontal conditions improved^[24]^. However, few studies have explored the correlation with visfatin expression levels in gingival tissues. Some researchers collected gingival tissue specimens from individuals with periodontal health and patients with periodontitis for immunohistochemical staining. The results indicated that gingival tissues of patients with periodontitis displayed widespread and strong visfatin expression, diffusely distributed within epithelial cells, human gingival fibroblasts, cytoplasm, endothelial cells, and the intercellular substance of human gingival fibroblasts, albeit weakly expressed^[25]^. This study suggested that localized visfatin expression in gingival tissues may be associated with the extent of periodontal inflammation.

In this study, we introduced an innovative approach by simultaneously collecting both GCF and gingival tissue specimens from the same periodontal site under varying periodontal conditions. We conducted separate assessments of their visfatin expression levels to explore the correlation between visfatin expression and the severity of periodontal inflammation. The primary objective of this study is to establish a theoretical foundation for utilizing visfatin as a potential biomarker for periodontitis.

**Methods**

**Study Population**

For this study, we selected patients admitted to the Stomatological Center of the First Affiliated Hospital of Fujian Medical University between October 2021 and June 2022. The study received approval from the Ethics Committee of the First Affiliated Hospital of Fujian Medical University (Aprove No: MRCTA, ECFAH of FMU [2021]155), and consent was obtained from all participating subjects.

Inclusion criteria: Participants of Han nationality, aged 18-70 years; Maintained consistent oral hygiene and dietary habits for at least 3 months; No history of prior periodontal treatment.

Exclusion criteria: Prolonged use of antibiotics, hormones, or immune-related medications; pregnancy or lactation; smoking habits; presence of systemic diseases that affect periodontal health, such as diabetes or autoimmune diseases.

In accordance with the aforementioned criteria and the classification criteria of the American Academy of Periodontology in 1999^[22]^, a total of 87 cases were included in the study. There were 43 cases in the chronic periodontitis group (CP group), 21 cases in the chronic gingivitis group (CG group), and 23 cases in the periodontal health group (PH group).

**Sample collection sites**

CP group: Teeth with periodontitis and no retentive value.

CG group: Third molars with no retentive value.

PH group: Third molars with no retentive value or teeth requiring gingivectomy during crown lengthening surgery.

**Clinical Examination and Data Collection**

General information about the subjects was collected, including gender, age, height, weight, and body mass index (BMI). Periodontal examinations are performed by the same professionally trained periodontist. A self-repeated measurement consistency test, requiring a kappa value >0.95, is performed prior to the examination. Periodontal indexes, including probing depth (PD), attachment loss (AL), plaque index (PLI)^[26]^, and bleeding index (BI)^[27]^, were to be recorded for each sample collection site in each group. Using the Williams periodontal probe, six sites (buccal: distal, central, mesial; and lingual: distal, central, mesial) on the selected teeth were checked. The average values of PD, AL, PLI, and BI were calculated.

**Collection and Detection of GCF**

To collect GCF, a sterile cotton ball was used to isolate the teeth from moisture. Carefully remove supragingival plaque, blowing gently with an air gun from the root to the crown of the tooth, which dries the surface of the tooth without blowing away the GCF. Absorbent paper points (DIA-ISO 0.02 030, Beijing Dayading Medical Appliance Co. LTD, China) were inserted into the mesial and distal gingival sulcus on the buccal side of the tooth. These paper points were removed after 30 seconds and placed in a sterile Eppendorf tube. If the absorbent paper points were contaminated with blood or saliva, they were discarded and resampled after 10 minutes. The paper points were sealed in Eppendorf tubes and stored in a -80°C refrigerator.

Visfatin levels in GCF were measured using a double-antibody one-step sandwich enzyme-linked immunosorbent assay (ELISA), following the instructions of the Human Visfatin ELISA Kit (Shanghai Qiaodu Biotechnology Co. LTD, China). Sensitivity is a minimum detection concentration of less than 1.0 ng/mL. Take out the required slats from the aluminum foil pouch after equilibrating at room temperature for 20 minutes, set up standard and sample wells, add 50 of different concentrations of standards to each standard well; add 10 μL of samples to be tested to the sample wells, then add 40 μL of sample dilution, and do not add to the blank wells; except for the blank wells, add 100 μL of horseradish peroxidase (HRP)-labeled detection antibody to each of the standard wells and sample wells, and seal the reaction wells with a plate-sealing membrane. The reaction wells were sealed with plate sealing membrane and incubated at 37℃ for 60 minutes; the liquid was discarded and patted dry on blotting paper, each well was filled with washing solution and left to stand for 1 minute, the washing solution was shaken off and the blotting paper was patted dry, and the plate was washed for 5 times; 50 μL of substrate A and B were added to each well, and the plate was incubated for 15 minutes at 37℃ away from light, and finally 50 μL of termination solution was added to each well. The absorbance value was measured with a microplate reader at a wavelength of 450 nm. A linear regression curve was constructed from the standard, and the concentration of each sample was calculated based on the curve equation.

**Gingival Tissues Collection, Immunohistochemical Staining, and Quantitative Analysis**

After GCF collection and periodontal examination, tooth extraction was performed. Small amounts of gingival tissue were cut from the buccal side of the extraction socket. These tissue samples were approximately 3~4 mm in length, and 1.5 mm in thickness, and included a full layer of epithelial and connective tissue. Gingival tissue samples were promptly fixed with 10% neutral formalin, embedded in paraffin, and made into 4 μm sections.

Paraffin sections were dewaxed and hydrated, and antigens were repaired with EDTA solution. An endogenous peroxidase blocker was added, incubated for 10 minutes, and rinsed with phosphate-buffered saline (PBS) (pH=7.4). The primary antibody (Recombinant Anti-Visfatin antibody [EPR21980], Abcam, UK) was added at a 1:2000 dilution ratio, incubated for 60 minutes, and rinsed with PBS. Secondary antibody (enzyme-labeled goat anti-mouse/rabbit IgG polymer) was applied, incubated for 15 minutes, and rinsed with PBS. Diaminobenzidine color development solution was added and incubated for 3 minutes. Finally, the sections were rinsed with distilled water, re-stained with hematoxylin for 1 minute, dehydrated, transparentized, and sealed. The experiment was conducted at an incubation temperature of 37°C, with consistent steps to ensure that different sections were treated in the same experimental environment and following the same procedure.

Microscope working conditions and shooting parameters were consistent. Five random fields of view (400 ×) were selected in three gingival tissue areas: full layer, epithelial layer, and connective tissue layer. An experimenter who was unaware of the section grouping captured all sections at once. Software (ImageJ, National Institutes of Health, USA) was used to quantitatively analyze the images. The gray value and optical density were converted and calibrated, and the average optical density (AOD) value of the positively stained area in the images was calculated. The average AOD values from the five fields of view represented the staining intensity of the area, indicating the expression level of the target protein^[28]^.

**Statistical Analysis**

Statistical analysis was carried out using statistical software (SPSS 26.0, IBM, USA). Mean ± standard deviation (Mean ± SD) was used to express the data's central tendency and dispersion. Dichotomous variables were compared using the χ2 test. The Mann-Whitney U test was employed to compare measurement data between the two groups. The Kruskal-Wallis H test and Bonferroni correction method were used for multiple post hoc comparisons. P<0.05 was considered statistically significant. Correlations between variables were assessed using Spearman's rank correlation analysis.

**Results**

**Comparison of General Information and Periodontal Indexes.**

When comparing the general information of the CP, CG, and PH groups, the CP group's age (49.14±9.49 years) was significantly higher than that of the CG group (25.29±5.07 years) and the PH group (26.87±4.58 years) (P<0.001). No statistically significant differences were observed in gender composition ratio, height, weight, or BMI among the three groups (P>0.05). During the selection of study subjects, the significant age difference between the CP, CG, and PH groups, where the CG and PH groups were predominantly younger, indicates a potential confounding effect of age on the study results. However, there are no studies that show a direct and specific correlation between age and visfatin. The study population was carefully selected to minimize the influence of confounding factors, ensuring balanced baseline characteristics among the three groups and resulting in reliable and comparable results.

Regarding the periodontal indexes in the CP, CG, and PH groups, the results indicated that PD, AL, PLI, and BI in the CP group were significantly higher than in the CG and PH groups, with statistically significant differences (P<0.001). PD and BI in the CG group were also higher than in the PH group, with statistically significant differences (P<0.002). While PLI in the CG group was higher than in the PH group, the difference was not statistically significant (P>0.05) (Table 1).

**Table 1.**

**Comparison of General Information and Periodontal Indexes in Different Periodontal Conditions**

|  | CP group | CG group | PH group | *χ²/K* | *P* |
| --- | --- | --- | --- | --- | --- |
|  | n =43 | n =21 | n =23 |  |  |
| Male (n) | 21 | 10 | 11 | 0.011 | 0.995 |
| Female (n) | 22 | 11 | 12 |  |  |
| Age (year) | 49.14±9.49 | 25.29±5.07 | 26.87±4.58 | 62.455 | <0.001* |
| Height (m) | 1.66±0.05 | 1.62±0.06 | 1.63±0.08 | 1.918 | 0.153 |
| Weight (Kg) | 65.19±5.90 | 62.71±4.83 | 62.48±7.50 | 1.949 | 0.149 |
| BMI(Kg/m^2^) | 23.74±1.96 | 23.74±2.07 | 23.32±1.47 | 0.542 | 0.763 |
| PD (mm) | 5.30±1.70 | 3.41±1.16 | 2.11±0.51 | 52.891 | <0.001* |
| AL (mm) | 5.69±1.97 | 0.00±0.00 | 0.00±0.00 | 74.102 | <0.001* |
| PLI | 2.40±0.82 | 1.29±0.72 | 0.65±0.78 | 42.050 | <0.001* |
| BI | 3.63±0.54 | 1.95±1.32 | 0.00±0.00 | 64.350 | <0.001* |

Data presented as Mean ±SD;

* Significant (p < 0.05).

**Comparison of Visfatin Expression in GCF and Gingival Tissues**

Visfatin levels in GCF for the CP, CG, and PH groups were 119.58±10.15 ng/mL, 93.85±6.81 ng/mL, and 74.64±11.37 ng/mL, respectively, and these differences were statistically significant (P<0.001). Statistically significant differences were also observed when comparing the three groups in pairs (P<0.001) (Figure 1A).

Immunohistochemical sections viewed under a light microscope revealed that visfatin was diffusely expressed in all layers of the epithelium, with the exception of cells in the stratum corneum and part of the granular layer adjacent to the stratum corneum. Visfatin staining was particularly intense in the basal layer. In the connective tissue layer, visfatin expression was observed in fibroblasts, endothelial cells, lymphocytes, and the intercellular matrix (Figure 2).

When comparing different periodontal conditions, the AOD values for the whole-layer field of view in the CP, CG, and PH groups were 0.52±0.10, 0.43±0.07, and 0.33±0.06, respectively. Significant differences were observed among the three groups, as well as in multiple comparisons (P<0.001) (Figure 1B).

Comparing different areas of expression, the AOD values for the epithelial layer and connective tissue layer in the CP group were 0.58±0.06 and 0.43±0.09, respectively. In the CG group, the AOD values for the epithelial layer and connective tissue layer were 0.45±0.04 and 0.37±0.09, respectively. In the PH group, the AOD values for the epithelial layer and connective tissue layer were 0.33±0.09 and 0.32±0.07, respectively. In the CP group, the epithelial layer had a higher AOD value than the connective tissue layer, and this difference was statistically significant (P<0.05). However, in the CG and PH groups, the AOD values for the epithelial layer were higher than those for the connective tissue layer, but the differences were not statistically significant (P>0.05) (Figure 1C). This suggests that the gingival tissues of patients in the CG and PH groups exhibited more balanced levels of visfatin expression between the epithelial and connective tissue layers.

**Figure 1.**

**Comparisons of Visfatin in GCF and Gingival Tissues**


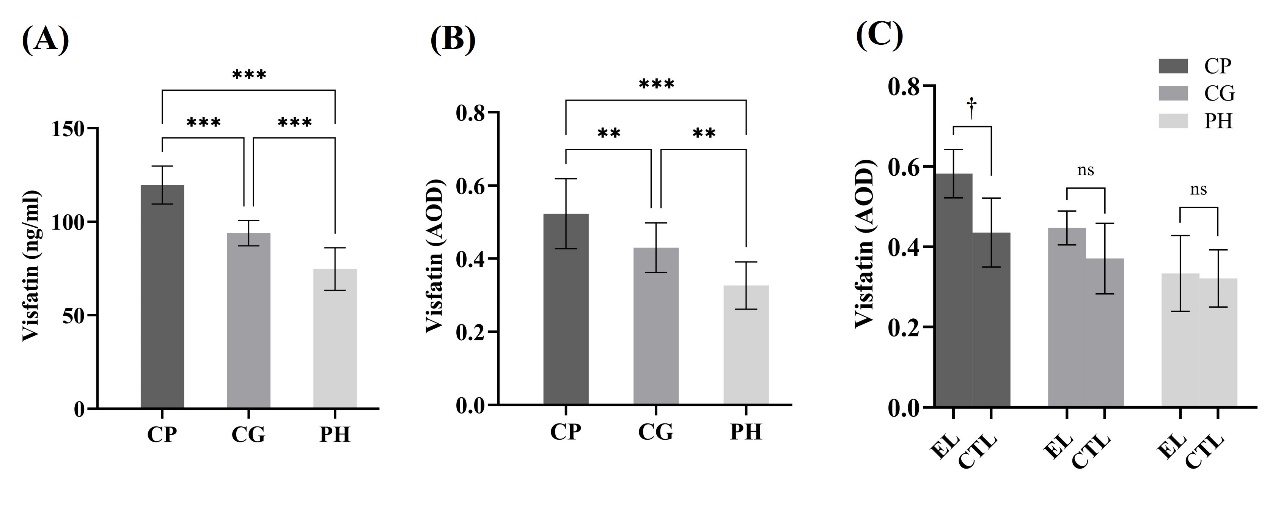


(A): Multiple comparisons of visfatin in GCF

(B): Multiple comparisons of visfatin in gingival tissues

(C): Comparison of visfatin expression in epithelial layer and connective tissue layer of gingival tissues

EL: Epithelial layer; CTL: Connective tissue layer.

Adjusted significance: * P < 0.0332; ** P < 0.0021; **** P < 0.0001;

† :Significant (p < 0.05);

ns: not significant.

**Figure 2.**

**Expression of Visfatin in Gingival Tissues of Different Periodontal Conditions**


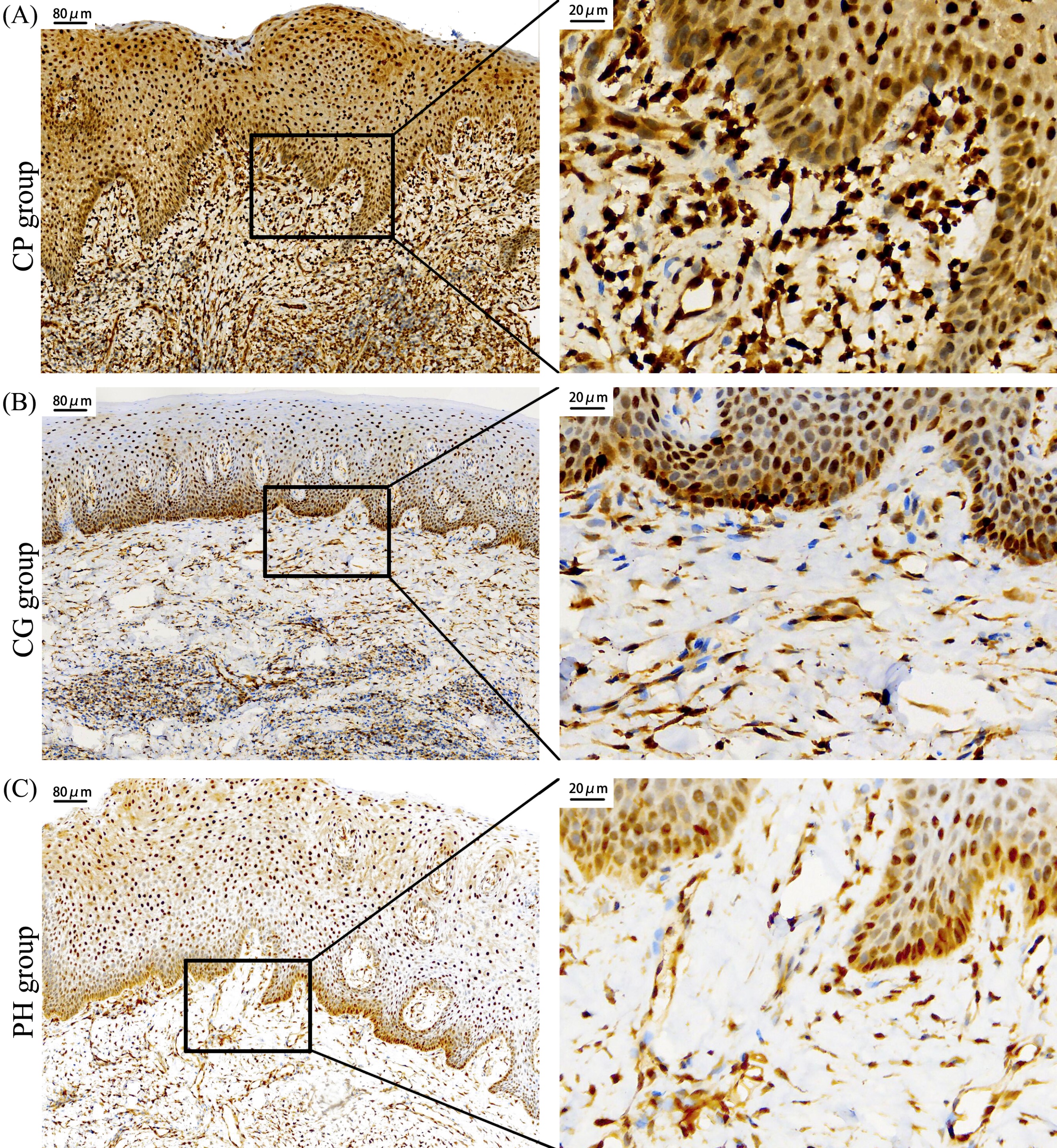


(A) CP group. (B) CG group. (C) PH group.

**Correlation between periodontal index and visfatin expression**

Spearman's correlation analysis was employed to assess the relationships between periodontal indexes (PD, AL, PLI, and BI), as well as the levels of visfatin in GCF and gingival tissues (Table 2). The results indicated that PD, AL, PLI, and BI were positively correlated with visfatin levels in GCF, with a statistically significant correlation (P<0.001). Similarly, PD, AL, PLI, and BI were positively correlated with visfatin levels in gingival tissues, also showing a statistically significant correlation (P<0.001). The correlation coefficients between visfatin levels in GCF and those in gingival tissues had a correlation coefficient of rs=0.772 (P<0.001).

**Table 2.**

**Correlation between Periodontal Indexes and Expression of Visfatin**

|  |  | PD | AL | PLI | BI | Visfatin(GCF) | Visfatin(Gingival) | |
| --- | --- | --- | --- | --- | --- | --- | --- | --- |
|  |  | (mm) | (mm) |  |  | (ng/ml) | (AOD) |  |
| Visfatin(GCF) | rs | 0.920* | 0.870* | 0.736* | 0.835* | 1.000 | 0.772* |  |
| (ng/ml) | p | <0.001 | <0.001 | <0.001 | <0.001 | - | <0.001 | |
| Visfatin(Gingival) | rs | 0.757* | 0.694* | 0.534* | 0.700* | 0.772* | 1.000 | |
| (AOD) | p | <0.001 | <0.001 | <0.001 | <0.001 | <0.001 | - | |

* Correlation is significant at the 0.01 level (2-tailed).

**Discussion**

The interaction between periodontal pathogenic bacteria and the host immune-inflammatory response is pivotal in the development and progression of periodontitis. Periodontal pathogens trigger the local secretion of inflammatory mediators and activate inflammatory signaling pathways in host cells to combat the pathogens, but this response also leads to bone resorption and collagen breakdown^[29]^. Periodontal pockets, clinical attachment loss, and gingival inflammation are key clinical manifestations of periodontitis, and the periodontal indices PD, AL, PLI, and BI objectively reflect the severity of periodontal inflammation^[30, 31]^. In this study, each periodontal index was consistently higher in the CP group compared to the CG and PH groups. PD and BI were also higher in the CG group compared to the PH group. This aligns with the expected order of periodontal destruction among the three groups.

Visfatin plays a role in this complex interaction of periodontal tissue destruction. Visfatin expression in GCF is strongly correlated with the periodontal condition. For example, Pradeep et al.^[32]^ showed that both localized visfatin levels in GCF and serum visfatin levels increased with disease severity, from the healthy control group to the gingivitis group, and finally to the periodontitis group. Visfatin levels in GCF and serum in the periodontitis group were positively correlated with periodontal indexes such as PD and AL. In this experiment, as the degree of periodontal lesions increased in the PH, CG, and CP groups, visfatin levels also increased significantly. There was a positive correlation between visfatin levels in GCF and periodontal indexes (PD, AL, PLI, BI), consistent with the findings of other studies^[23, 24, 32]^. GCF and serum visfatin levels increased in both periodontitis and gingivitis patients compared to healthy controls, and the mean salivary visfatin levels also increased with the severity of periodontal tissue destruction^[33, 34]^. Non-surgical periodontal treatment significantly improved the periodontal conditions of patients with periodontitis, leading to reduced PD, increased clinical attachment levels, and decreased release of inflammatory mediators from local tissues. After periodontal treatment, GCF and serum visfatin levels were substantially reduced, changing in parallel with the degree of periodontal destruction and influenced by periodontal interventions^[23]^. In summary, GCF and serum visfatin levels were closely associated with the severity of periodontal inflammation, and the changes in both were consistent.

Visfatin expression levels in gingival tissues are also closely linked to periodontal conditions. Elevated visfatin expression has been observed in inflamed gingival tissues in both humans and mice^[15]^. Immunohistochemical staining revealed higher levels of visfatin in aggressive and chronic periodontitis gingival tissues compared to healthy gingival tissues, with no difference between aggressive and chronic periodontitis groups^[35]^. Visfatin expression was significantly higher in gingival tissues of periodontitis patients compared to those of healthy individuals, suggesting that local synthesis of visfatin in inflamed gingival tissues may contribute to increased visfatin levels in gingival tissues and serum^[36]^. In this experiment, we compared the expression levels of visfatin in the entire field of view of gingival tissues. Notably, the intensity of visfatin expression progressively weakened in the CP, CG, and PH groups. Furthermore, our correlation analysis revealed a positive association between periodontal indexes, including PD, AL, PLI, BI, and visfatin levels within gingival tissues. As the severity of periodontal destruction increased, so did the expression of visfatin in gingival tissues.

Additionally, our correlation analysis unveiled a positive relationship between visfatin levels in GCF and visfatin levels in gingival tissues. GCF, being a complex mixture originating from serum and periodontal tissues, is widely acknowledged as a reflection of the inflammatory state of periodontal tissues^[37]^. This finding leads us to speculate that GCF might, in part, originate from the gingival tissue itself, signifying its role in the local inflammatory context.

The distribution sites of visfatin expression in gingival tissues vary across different periodontal conditions. Studies have shown that visfatin is strongly expressed in all layers of gingival tissue in patients with periodontitis, with diffuse distribution in the epithelial layer and expression in fibroblasts, endothelial cells, and intercellular matrix in the connective tissue layer^[35, 36]^. In healthy gingiva, visfatin is mainly limited to the basal and parabasal layers of the epithelium, with lower intensity elsewhere and weak or no expression in the connective tissue layer^[25, 35]^. Some studies have reported variations, such as no positive staining in the basal layer of gingival epithelium in gingivitis and healthy control patients. A small amount of visfatin was expressed in the connective tissue layer of gingival tissues in patients with gingivitis, while no expression of visfatin was found in the connective tissue layer of gingival tissues in healthy individuals^[36]^. The distribution of visfatin expression within gingival tissues exhibits slight variations. The results of this experiment indicate that in the CP group, the level of visfatin in the epithelial layer surpasses that in the connective tissue layer. In contrast, the CG and PH groups display a balanced expression of visfatin in both the epithelial and connective tissue layers, with no significant differences. Numerous studies mentioned in the literature consistently report a higher expression of visfatin in the epithelial layer compared to the connective tissue layer. This phenomenon was also observed within the CP group in our study. The authors posit that the observed differences in expression distribution across a limited number of studies may arise from variations in the antibody products utilized. Moreover, the elevated level of visfatin in the epithelial layer compared to the connective tissue layer in the CP group may be attributed, in part, to certain epithelial cells demonstrating a greater propensity or capacity to produce visfatin. Additionally, it could be influenced by the anatomical positioning of the gingival and sulcus epithelium, making them more susceptible to stimulation from the oral flora, particularly periodontal pathogenic bacteria, potentially leading to enhanced visfatin production.

Periodontal pathogenic bacteria like *Porphyromonas gingivalis* and *Clostridium nucleatum*, as well as pro-inflammatory cytokines like IL-1β, can stimulate visfatin synthesis in periodontal ligament cells and gingival fibroblasts, with increased synthesis under inflammatory and infectious conditions^[36, 38]^. Visfatin mediates the inflammatory response by activating nuclear factor-κB and phosphatidylinositol trihydroxykinase signaling pathways, which inhibit neutrophil apoptosis^[39]^. Visfatin may play a role in periodontitis by upregulating MMP-1 and chemokine-2 in periodontal ligament cells^[40]^ . It can upregulate pro-inflammatory cytokines and matrix metalloproteinases in various cell types, leading to connective tissue and periodontal bone loss^[25]^. Visfatin plays a crucial role in the resorptive remodeling of alveolar bone and is increased in inflammatory diseases involving bone resorption, such as rheumatoid arthritis and osteoarthritis^[41, 42]^. Combined with the experimental results, it can be speculated that periodontal pathogenic bacteria might damage periodontal tissues through the upregulation of visfatin expression in local gingival tissues and GCF, further regulating various inflammatory factors. Periodontal pathogenic bacteria can cause inflammation in periodontal tissues, and periodontal indexes like PD, AL, and BI reflect the severity of periodontal inflammation. Simple gingival inflammation without periodontal pockets and attachment loss can also lead to elevated levels of GCF and visfatin in gingival tissues. The CG group exhibited less severe periodontal inflammation than the CP group, resulting in lower levels of GCF and visfatin in gingival tissues due to reduced exposure to periodontal pathogenic bacteria and their virulence products. More inflammation in periodontal tissues leads to higher visfatin synthesis in GCF and gingival tissues, resulting in a stronger destructive effect.

This study also has limitations. This experiment is a cross-sectional study, which has not yet been able to directly reveal the direct causal relationship and specific mechanisms between visfatin expression levels and periodontal disease. Second, the inclusion and exclusion criteria of this experiment were more stringent, limiting the sample size, but this reduces a variety of potential confounders such as systemic diseases. Also, we only analyzed a single inflammatory factor, which does not yet fully reflect the overall situation of GCF and gingival tissue inflammation in patients with periodontitis. However, the conclusions drawn in this paper, when combined with the literature analysis, provide a basis for the prevention and treatment of periodontitis. In the future, larger samples and longitudinal studies are needed to further investigate the relationship between visfatin and the pathogenesis of periodontitis

**Conclusions**

Visfatin in GCF and gingival tissues appears to collaborate in damaging periodontal tissues, and it plays a role in the pathogenesis of periodontitis. Visfatin serves as a potential biomarker for periodontitis and may contribute to its pathogenesis.

**List of abbreviations**

| gingival crevicular fluid | GCF |
| --- | --- |
| chronic periodontitis | CP |
| chronic gingivitis | CG |
| periodontal health | PH |
| interleukin-1β | IL-1β |
| body mass index | BMI |
| probing depth | PD |
| attachment loss | AL |
| plaque index | PLI |
| bleeding index | BI |
| enzyme-linked immunosorbent assay | ELISA |
| phosphate-buffered saline | PBS |
| Epithelial layer | EL |
| Connective tissue layer | CTL |

**Declarations**

**Ethics approval and consent to participate**

The study received approval from the Ethics Committee of the First Affiliated Hospital of Fujian Medical University(No. MRCTA, ECFAH of FMU [2021]155). Informed consent was obtained from all the participants.

**Consent for publication**

Not applicable.

**Availability of data and materials：**

The data that support the findings of this study are available from the corresponding author upon reasonable request.

**Competing interests:**

The authors declared no potential conflicts of interest concerning the research, authorship, and/or publication of this article.

**Funding:**

Natural Science Foundation of Fujian Province, China (Grant No. 2021J01223 and No. 2021J01215)

**Authors' contributions:**

The study was designed and conducted by YW. Most of the experiments and experimental data analysis were completed by KX and LC, while YM, HB, WC and XL participated in some experiments. KX and LC participated in the writing of the first draft, and the final revision of the manuscript was completed by YW. KX and LC both contributed equally to this study. All authors read and approved the final manuscript.

**Acknowledgements:**

We would like to thank the students of the School of Stomatology of Fujian Medical University for their help in the experiment.

**References**

[1] Kassebaum N J, Bernabé E, Dahiya M, Bhandari B, Murray C J, Marcenes W. Global burden of severe periodontitis in 1990-2010: a systematic review and meta-regression [J]. J Dent Res, 2014, 93(11): 1045-53.

[2] Armitage G C. Periodontal diagnoses and classification of periodontal diseases [J]. Periodontol 2000, 2004, 34: 9-21.

[3] Mopidevi A, Penmetsa G S, Dwarkanath C D, Dubba K, Gadde P. Salivary visfatin concentrations in patients with chronic periodontitis: An analysis before and after periodontal therapy [J]. Indian J Dent Res, 2019, 30(6): 864-9.

[4] Tabari Z A, Azadmehr A, Nohekhan A, Naddafpour N, Ghaedi F B. Salivary visfatin concentrations in patients with chronic periodontitis [J]. J Periodontol, 2014, 85(8): 1081-5.

[5] Balli U, Ongoz Dede F, Bozkurt Dogan S, Gulsoy Z, Sertoglu E. Chemerin and interleukin-6 levels in obese individuals following periodontal treatment [J]. Oral Dis, 2016, 22(7): 673-80.

[6] Klöting N, Blüher M. Adipocyte dysfunction, inflammation and metabolic syndrome [J]. Rev Endocr Metab Disord, 2014, 15(4): 277-87.

[7] Fukuhara A, Matsuda M, Nishizawa M, Segawa K, Tanaka M, Kishimoto K, Matsuki Y, Murakami M, Ichisaka T, Murakami H, Watanabe E, Takagi T, Akiyoshi M, Ohtsubo T, Kihara S, Yamashita S, Makishima M, Funahashi T, Yamanaka S, Hiramatsu R, Matsuzawa Y, Shimomura I. Visfatin: a protein secreted by visceral fat that mimics the effects of insulin [J]. Science, 2005, 307(5708): 426-30.

[8] Wnuk A, Stangret A, Wątroba M, Płatek A E, Skoda M, Cendrowski K, Sawicki W, Szukiewicz D. Can adipokine visfatin be a novel marker of pregnancy-related disorders in women with obesity? [J]. Obes Rev, 2020, 21(7): e13022.

[9] Jiang Y K, Deng H Y, Qiao Z Y, Gong F X. Visfatin level and gestational diabetes mellitus: a systematic review and meta-analysis [J]. Arch Physiol Biochem, 2021, 127(5): 468-78.

[10] Dakroub A, Nasser S A, Kobeissy F, Yassine H M, Orekhov A, Sharifi-Rad J, Iratni R, El-Yazbi A F, Eid A H. Visfatin: An emerging adipocytokine bridging the gap in the evolution of cardiovascular diseases [J]. J Cell Physiol, 2021, 236(9): 6282-96.

[11] Romacho T, Sánchez-Ferrer C F, Peiró C. Visfatin/Nampt: an adipokine with cardiovascular impact [J]. Mediators Inflamm, 2013, 2013: 946427.

[12] Franco-Trepat E, Alonso-Pérez A, Guillán-Fresco M, Jorge-Mora A, Gualillo O, Gómez-Reino J J, Gómez Bahamonde R. Visfatin as a therapeutic target for rheumatoid arthritis [J]. Expert Opin Ther Targets, 2019, 23(7): 607-18.

[13] Bayani M, Heidari M, Almasi-Hashiani A. Periodontal disease and visfatin level: A systematic review and meta-analysis [J]. PLoS One, 2023, 18(11): e0293368.

[14] Zhu J, Zhang S, Shi J, Ning N, Wei Y, Zhang Y. Periodontitis is associated with the increased levels of visfatin: a meta-analysis [J]. BMC Oral Health, 2023, 23(1): 799.

[15] Park K H, Kim D K, Huh Y H, Lee G, Lee S H, Hong Y, Kim S H, Kook M S, Koh J T, Chun J S, Lee S E, Ryu J H. NAMPT enzyme activity regulates catabolic gene expression in gingival fibroblasts during periodontitis [J]. Exp Mol Med, 2017, 49(8): e368.

[16] Ghallab N A. Diagnostic potential and future directions of biomarkers in gingival crevicular fluid and saliva of periodontal diseases: Review of the current evidence [J]. Arch Oral Biol, 2018, 87: 115-24.

[17] Alarcón-Sánchez M A, Heboyan A, Fernandes G V O, Castro-Alarcón N, Romero-Castro N S. Potential Impact of Prosthetic Biomaterials on the Periodontium: A Comprehensive Review [J]. Molecules, 2023, 28(3).

[18] Fatima T, Khurshid Z, Rehman A, Imran E, Srivastava K C, Shrivastava D. Gingival Crevicular Fluid (GCF): A Diagnostic Tool for the Detection of Periodontal Health and Diseases [J]. Molecules, 2021, 26(5): 1208.

[19] Almehmadi A H, Alghamdi F. Biomarkers of alveolar bone resorption in gingival crevicular fluid: A systematic review [J]. Arch Oral Biol, 2018, 93: 12-21.

[20] Madureira D F, Lucas De Abreu Lima I, Costa G C, Lages E M B, Martins C C, Aparecida Da Silva T. Tumor Necrosis Factor-alpha in Gingival Crevicular Fluid as a Diagnostic Marker for Periodontal Diseases: A Systematic Review [J]. J Evid Based Dent Pract, 2018, 18(4): 315-31.

[21] Alarcón-Sánchez M A, Guerrero-Velázquez C, Becerra-Ruiz J S, Rodríguez-Montaño R, Avetisyan A, Heboyan A. IL-23/IL-17 axis levels in gingival crevicular fluid of subjects with periodontal disease: a systematic review [J]. BMC Oral Health, 2024, 24(1): 302.

[22] Armitage G C. Development of a classification system for periodontal diseases and conditions [J]. Ann Periodontol, 1999, 4(1): 1-6.

[23] Türer Ç C, Balli U, Güven B, Çetinkaya B, Keleş G. Visfatin levels in gingival crevicular fluid and serum before and after non-surgical treatment for periodontal diseases [J]. J Oral Sci, 2016, 58(4): 491-9.

[24] Raghavendra N M, Pradeep A R, Kathariya R, Sharma A, Rao N S, Naik S B. Effect of non surgical periodontal therapy on gingival crevicular fluid and serum visfatin concentration in periodontal health and disease [J]. Dis Markers, 2012, 32(6): 383-8.

[25] Yao S, Jiang C, Zhang H, Gao X, Guo Y, Cao Z. Visfatin regulates Pg LPS-induced proinflammatory/prodegradative effects in healthy and inflammatory periodontal cells partially via NF-κB pathway [J]. Biochim Biophys Acta Mol Cell Res, 2021, 1868(8): 119042.

[26] Silness J, Loe H. Periodontal disease in pregnancy .II. correlation between oral hygiene and periodontal condition [J]. Acta Odontol Scand, 1964, 22: 121-35.

[27] Mazza J E, Newman M G, Sims T N. Clinical and antimicrobial effect of stannous fluoride on periodontitis [J]. J Clin Periodontol, 1981, 8(3): 203-12.

[28] Fang Q, Zheng S, Chen Q, Chen L, Yang Y, Wang Y, Zhang H, Chen J. The protective effect of inhibiting mitochondrial fission on the juvenile rat brain following PTZ kindling through inhibiting the BCL2L13/LC3 mitophagy pathway [J]. Metab Brain Dis, 2023, 38(2): 453-66.

[29] Graves D T, Cochran D. The contribution of interleukin-1 and tumor necrosis factor to periodontal tissue destruction [J]. J Periodontol, 2003, 74(3): 391-401.

[30] Newbrun E. Indices to measure gingival bleeding [J]. J Periodontol, 1996, 67(6): 555-61.

[31] Beltrán-Aguilar E D, Eke P I, Thornton-Evans G, Petersen P E. Recording and surveillance systems for periodontal diseases [J]. Periodontol 2000, 2012, 60(1): 40-53.

[32] Pradeep A R, Raghavendra N M, Prasad M V, Kathariya R, Patel S P, Sharma A. Gingival crevicular fluid and serum visfatin concentration: their relationship in periodontal health and disease [J]. J Periodontol, 2011, 82(9): 1314-9.

[33] Amita C, Neethu R, Anirban C, Irfanulla K M. The Role of Visfatin (Adipocytokine) Biomarker in Oral Health and Diseases among Nonobese Indian Population: A Proteomic Assay [J]. Global medical genetics, 2021, 8(3): 104-8.

[34] Greeshma S, Seba A, Aparna N, M R. Comparative evaluation of salivary visfatin levels in healthy and periodontally diseased patients before and after scaling and root planing [J]. Journal of Pharmacy And Bioallied Sciences, 2021, 13(5): S624-28.

[35] Tabari Z A, Keshani F, Sharbatdaran M, Banishahabadi A, Nejatifard M, Ghorbani H. Visfatin expression in gingival tissues of chronic periodontitis and aggressive periodontitis patients: An immunohistochemical analysis [J]. Dent Res J (Isfahan), 2018, 15(2): 104-10.

[36] Damanaki A, Nokhbehsaim M, Eick S, Götz W, Winter J, Wahl G, Jäger A, Jepsen S, Deschner J. Regulation of NAMPT in human gingival fibroblasts and biopsies [J]. Mediators Inflamm, 2014, 2014: 912821.

[37] Bostanci N, Belibasakis G N. Gingival crevicular fluid and its immune mediators in the proteomic era [J]. Periodontol 2000, 2018, 76(1): 68-84.

[38] Nogueira A V, Nokhbehsaim M, Eick S, Bourauel C, Jäger A, Jepsen S, Cirelli J A, Deschner J. Regulation of visfatin by microbial and biomechanical signals in PDL cells [J]. Clin Oral Investig, 2014, 18(1): 171-8.

[39] Özcan E, Saygun N I, Ilıkçı R, Karslıoğlu Y, Muşabak U, Yeşillik S. Increased visfatin expression is associated with nuclear factor-kappa B and phosphatidylinositol 3-kinase in periodontal inflammation [J]. Clin Oral Investig, 2017, 21(4): 1113-21.

[40] Nokhbehsaim M, Eick S, Nogueira A V, Hoffmann P, Herms S, Fröhlich H, Jepsen S, Jäger A, Cirelli J A, Deschner J. Stimulation of MMP-1 and CCL2 by NAMPT in PDL cells [J]. Mediators Inflamm, 2013, 2013: 437123.

[41] Laiguillon M C, Houard X, Bougault C, Gosset M, Nourissat G, Sautet A, Jacques C, Berenbaum F, Sellam J. Expression and function of visfatin (Nampt), an adipokine-enzyme involved in inflammatory pathways of osteoarthritis [J]. Arthritis Res Ther, 2014, 16(1): R38.

[42] Gosset M, Berenbaum F, Salvat C, Sautet A, Pigenet A, Tahiri K, Jacques C. Crucial role of visfatin/pre-B cell colony-enhancing factor in matrix degradation and prostaglandin E2 synthesis in chondrocytes: possible influence on osteoarthritis [J]. Arthritis Rheum, 2008, 58(5): 1399-409.
